# Death registration and certification in Ghana

**DOI:** 10.4314/gmj.v59i3.1

**Published:** 2025-09

**Authors:** Edwin K Wiredu

**Affiliations:** Department of Pathology, University of Ghana Medical School, Accra, Ghana

Death registration and certification are inexorably connected, as death certification provides the data that form the bedrock for accurate death registration. In Ghana, registration of all deaths is required by law, governed by the Registration of Births and Deaths Act 2020 (Act 1027).^1^ Sections 29, 30 and 31 of Act 1027 deal directly with certificates that are issued after the death of a person. Under section 29, the medical practitioner who was in attendance during the illness of the deceased is required to issue a medical certificate that states the particulars of the death, including the cause of death (called the Medical Certificate of Cause of Death or MCCD). Where a health practitioner was in attendance during the illness of the deceased, the supervising medical practitioner shall issue the medical certificate that states the cause of death. It is quite clear here that the MCCD can only be issued by a medical practitioner (registered as such under the Health Professions Regulatory Bodies Act 2013, Act 857, Part 2, Sections 29, 30 and 31^2^). A Physician Assistant does not qualify under Act 857 to issue an MCCD. Act 1027 empowers the District Registrar to issue a Death Certificate in the prescribed form after payment of the prescribed fee.

Where, for medico-legal reasons, the death is required by the Coroner's Act of 1960 (18)^3^ to be reported to the Coroner, the Coroner, under Section 30 of Act 1027, shall complete and sign a certificate that states the cause of the death (Certificate of Cause of Death). This certificate is submitted to the District Registrar (of Births and Deaths), who then registers the particulars of the deceased in the register of deaths.

The purpose of these legal provisions is to ensure complete registration of all deaths in the country. The MCCD is a very important document as it provides the majority of the data on causes of death and is an essential and fundamental component of civil registration and vital statistics (CRVS) services. In recent times, the Births and Deaths Registry (BDR) has undertaken numerous workshops and activities to strengthen the country's CRVS system. Due to poor coverage and non-registration of deaths, and despite these laws, only 39% of all estimated deaths were reported and captured by the BDR in 2023, which was 5.2% higher than in 2022.^4^ A new Strategic Plan for the CRVS system is underway, and it is expected to improve the system substantially. However, the issues of the MCCD do not lie directly with the BDR but with the medical establishment.

The current MCCD form recommended by the World Health Assembly is the international form of medical certificate of cause of death^5^ (International MCCD), which is a standardised form used to document the cause of death (COD). The form is in three sections. The first section, labelled Administrative Data, covers particulars of the deceased person. The second, called Frame A, is the same worldwide and contains the COD information.^6^ This section is in two parts, Part 1 and Part 2. Part 1 is used to describe sequentially and in a causally-related manner the morbid events leading to the death of the deceased, stating the time interval between the onset of each condition and the date of death. Part 2 is used for conditions that do not belong in Part 1 (not causally related to the events stated in Part 1) but whose presence contributed to death. Frame B contains separate detailed questions such as previous surgery, mode of death or place of occurrence of death^6^ and provides additional information necessary to code correctly the underlying cause of death. The form of MCCD prescribed by law in Ghana (LI2436, Registration of Births and Deaths Regulations, 2021) 7 and called Form 29 is a modification of the International MCCD. Form 29 contains three sections that do not include Frame B of the International MCCD. The first section contains a declaration of having attended the deceased by the certifying medical practitioner and the particulars of the deceased. The second section is the statement of COD and is identical to Frame A of the International MCCD. The final section contains the particulars of the attending/certifying medical practitioner.

In this issue of the Ghana Medical Journal, Agyekum et al^8^ report on the assessment of errors in death certification and mortality patterns and trends among medical patients at Korle Bu Teaching Hospital from 2019 to 2023. They discuss the importance of the MCCD to the family of the deceased and the relevance of the information in it to national development, which will not be discussed in this commentary. Nevertheless, it is necessary to state here that due to the significant role of death statistics in national planning, it is critically important that accurate and reliable information about the diseases leading to death is recorded. Errors in recording COD undermine the credibility and validity of the data that form the evidence base for public health policy development and planning.

Acceptable (gold standard) medical cause of death certification (i.e. proper completion of the MCCD) is absolutely dependent on accurately recording the events leading to death in an acceptable sequence, that is easy to read, and without the use of nonstandard abbreviations, symptoms, modes of dying and other ill-defined causes that can make coding very difficult.^9^ Errors in MCCDs are common and can range from minor omissions to major inaccuracies, affecting the reliability of mortality data used for public health planning and policy-making. Error frequencies range from 17.7% to 96% in hospital-based studies and occur even at institutions where autopsy pathologists trained in death certification are available resources.^10^

One study found an error in all 1603 certificates studied^11^ while a scoping review of all published articles in India between December 31, 1998 and December 31, 2020, reported up to 100% incomplete medical certification of cause of death.^12^ Errors in filling MCCDs are generally grouped into major and minor based on their impact on correctly identifying the underlying cause of death (UCOD)^11^-^13^, which is the only registrable item on the MCCD.

Most studies accept the following as Major Errors:

These fundamentally undermine the accuracy of the UCOD.

**Unacceptable UCOD:** Listing mechanisms (e.g., cardiac arrest, respiratory failure) or ill-defined conditions (e.g., senility, symptoms) as the UCOD without a specific antecedent cause.

**Improper Sequence of Events:** Mis-ordering the relationships between the causes of death listed on the certificate or wrong sequencing of the events directly leading to death.

**Incompatible Causal Relationship:** Listing two or more unrelated or competing causes of death.

**Incomplete Information:** Critical information in Part I, such as the specific cause of death, is missing

**Wrong Manner of Death:** Incorrectly classifying the cause of death (e.g., accident classified as natural).

Minor Errors consist of technical or clerical issues that do not directly impact the identification of the UCOD but reduce the overall quality of the certification.

**Abbreviations:** Using abbreviations in the cause of death sections.

**Missing Time Intervals:** Not including the time between the onset of a condition and death for the causes listed.

**Technical or Clerical Errors:** Incorrect or missing personal identifiers, incomplete physician details.

Illegible handwriting was considered major in one paper, as it potentially can render the MCCD unsuitable for statistical purposes if the underlying cause could not be determined^13^ while in another, it was considered minor.^10^

These inconsistencies make critical comparisons between different studies inaccurate and misleading. Illegible handwriting should, therefore, be further clarified in future studies as:

Major

**Illegible handwriting:** Critical information in Part I, such as the specific cause of death, is illegible.

Minor

**Illegible handwriting:** Entries that are difficult to read but do not directly impact the UCOD.

Some systems use a scale to grade errors by severity, such as a **0-IV**^14^ or **0-V**^10^ scale. Other Studies categorise errors by category into **Part I Errors**, comprising major errors found in Part I, such as an unacceptable UCOD or improper sequencing and **Part II & Technical Errors** made up of minor errors relating to Part II (other significant conditions) or technical aspects of the form, like using abbreviations or having illegible handwriting.^15^

The World Health Organisation (WHO) considers listing more than one COD on a single line in Part I of an MCCD to be a major error, as Part I is designed to record a sequential chain of events leading to death, with each cause listed on a separate line so that the underlying cause and the causal sequence can be identified. Listing multiple causes on one line disrupts this intended sequence and can lead to misclassification of the underlying cause of death. However, writing a single underlying cause of death can be very challenging in complex cases with multiple co-morbidities that result in multiple causal pathways.^15^ WHO has provided an international coding standard that is applied to all causes reported on the MCCD, either to endorse the reported UCOD or select a more appropriate alternative to be used for statistical reporting and international comparison.^16^ The UCOD using this standardised method alone may not adequately describe the pathologic processes responsible for most deaths, and thus potentially understate the importance of other significant contributing causes of death.^16^

In their paper, Agyekum et al. compared their results with two earlier local studies, Akapko et al. and Ossei et al.^8^ These earlier studies used data from the old MCCD form, which comprised only Frame A of the current WHO-recommended form. The information obtained in these two studies would therefore be quite different, making direct comparisons misleading. For example, ticking or selecting the wrong mode or manner of death will be a major error using the current form, but will not occur using the old form because it was not a required field.

While the high error rate of 76.65% in stating the underlying cause of death is worrying for our CRVS data, the serious medico-legal breaches suggested as the causes are more worrying. If there is uncertainty about the underlying cause of death for whatever reason, the certifying doctor is constrained by the Coroner's Act from issuing an MCCD. The use of a Resident with insufficient knowledge of the deceased's history or who was not directly involved in the patient's care also raises legal issues. The first part of the Ghana MCCD contains a declaration to be made by the certifying medical practitioner of having medically attended to the deceased before commencing to fill in the rest of the certificate.^7^ It is not only important but imperative that supervising consultants and trainers act immediately to correct these practices.

The problem of poor filling of the MCCD forms in Korle Bu Teaching Hospital may not be limited only to the Departaient of Medicine and Therapeutics. Training in completing the MCCD is important in reducing errors^12^ and, as suggested by Agyekum et al.^8^, should be incorporated into the training of all Residents. This could be put in the training logbook (with the concurrence of the relevant Medical Colleges) with successful completion of a minimum number of MCCD forms as a competency outcome. Targeted training programmes involving a comprehensive training for doctors on how to correctly complete MCCDs as part of their orientation after employment/posting should be established by hospitals. Alternatively, hospitals may implement a training of trainers (TOT) strategy where key medical staff are trained who, in turn, provide continuous training of doctors.^13^ This can be done as a half-day to full-day MDC-accredited CPD programme.

Establishing systematic processes to review and provide feedback on completed MCCDs can improve quality and serve as an additional means of ameliorating the situation. Incomplete or poor-quality medical records can make it difficult for certifying doctors to accurately assign causes of a death, particularly when information on events on the day of death is poor or lacking, as the study suggested. Hospitals should seriously consider setting up standardised procedures for auditing and reporting on the quality of patients' notes to improve standards. It would be prudent and justifiable for the Ministry of Health, working with the BDR, to develop policy guidelines to direct all Health Facilities in the accurate filling of the MCCD.
